# The General Infirmary, Gloucester

**Published:** 1890-04-19

**Authors:** 


### The Hospital Workshop.

(By our Special Commissioner.)

### No. XXV.—The General Infirmary, Gloucester.

A plain substantial brick building—in keeping with the ancient cathedral town in which it stands—constitutes the head-quarters of this institution. Fronted by an ample lawn space, it faces Southgate Street. On the left, by the porter's lodge, is the Eye Hospital, which some years ago was moved hither from another part of the town, and the two charities brought under one management. The house surgeon, Mr. J. F. Wood, kindly received and gave every information to your commissioner on the occasion of a late visit.

The hospital has a hundred and fifty-six beds, but lately several wards have been closed through lack of funds. Admission is freely given to all cases of emergency and accident, or to patients requiring operation. Poor people suffering from acute disease are taken in on the guarantee of any resident minister of religion. With these exceptions applicants must have a subscriber's recommendation. There are no paying patients, but each govenor is entitled to recommend additional in-patients by payment of ten shillings weekly, or five shillings for out-patients. The committee seems specially anxious to exclude undeserving cases. A notice heads the report to the effect: "It is earnestly requested that every subscriber should inform himself of the circumstances of the person he recommends, as several have been admitted to the infirmary able to have paid for their case, whilst others have endeavoured to get admission whose circumstances have been known to be good." The subscriber, moreover, has to state his belief that the applicant is poor and a real object of charity. Of the patients many come from Gloucester itself or from neighbouring towns, others from Wales and the Forest of Dean, while a large number is derived from the surrounding agricultural districts. The ophthalmic branch seems to be specially useful, as there is no other institution of the kind nearer than Bristol. That its advantages are recognised is shown by the fact that during the year 1888 there were 152 in, and 1,106 out-patients.

The wards in the main building are lofty and comfortable, containing, on an average, about thirteen beds apiece. The floors are of unpolished oak. Heating is effected by means of open fireplaces. The bedsteads are of iron with hair-stuffed beds and white coverlets. In the "female surgical" was a boy of five, who had been crushed by the branch of a falling tree. One thigh was broken and deeply wounded, while the bones of the opposite leg were also fractured. These details were learnt from the nurse, for the child was taken with a fit of shyness and could not be induced to give any account of his accident. Near by was a girl with spinal curvature waiting to be fitted with a plaster of Paris jacket after the method introduced by Professor Sayre, of New York, and now very generally adopted in such cases. In the next bed was a girl recovering from "pleurisy with effusion," which had developed in the hospital. She had been admitted in the first place to be treated for "patellar bursitis," or what is commonly known as "housemaid's knee."

Connected with the main block by a passage is a new wing containing large out-patient and consulting rooms, surgery, dispensary, and male accident ward on the ground floor. The practice is here followed of putting up fractures at once in plaster of Paris. If there be much swelling a "Croft's splint" is used, which can be split open over the seat of the effusion. One case was a damaged kidney, resulting from a football accident, a class of injury comparatively frequent of late years since that game has become popular. One of the patients has paralysis of the "deltoid," or large muscle of the shoulder, from an injury to the "circumflex" nerve following dislocation. Treatment by galvanism appears to be effecting some improvement. Outside this ward is a lift to the operating room. The latter seems exceptionally well adapted to its purpose. A large skylight in the roof is continuous with a side window, the result being a very perfect light. For night operations a Wenham burner hangs directly over the operating table. The instruments are ranged in labelled shelves within a glass case, and are thus readily available at a moment's notice. Near the operating-room is one of the closed wards. The beds are wrapped up in a ghostly fashion in white sheets, and the deserted look of the place presents a vivid contrast with the occupied wards. The management of the hospital appears to be efficient and economical, and, therefore, the closing of these wards through want of funds casts no little reproach upon the county. Among the female medical cases was a woman of thirty-two, admitted the day before, with profuse hæmorrhage from the stomach, due to an ulcer. She is a dressmaker, an occupation specially prone to the affection named, and almost always accompanied by marked anæmia or "poverty of blood." In the male medical ward is a waterman, forty-three years of age, suffering from malignant tumours in the neck. He first came under treatment twelve months ago, and was re-admitted a few weeks since with great difficulty in swallowing. That symptom has so far improved that he is now able to take food fairly well, but his look is fatally suggestive of advanced malignant disease. Near him is a corn porter of forty-one, prostrate with a large aneurism in the chest. He has been under treatment several times before with relief on each occasion. His present condition is most precarious, for the breathing is laboured and general œdema has involved the lower limbs. A young man, a waiter, in the same ward, is wasted to a shadow with acute phthisis or "galloping consumption." There have been profuse night sweats, which belladonna has somewhat checked. His temperature has ranged between 100 degrees F. and 104 degrees F., and once touched 105. He has been in for a fortnight and the disease, so far as can be traced, showed itself only five weeks since.

Students or qualified men on payment of a fee are allowed to enter here as residents. The field offered in provincial hospitals seems to be hardly yet appreciated at its proper value. When there are no resident clerks the whole of the work falls on the house surgeon, a task which it is obviously unfair to throw on the shoulders of one man. Nurses' accommodation is fairly good, but might be improved. The report is clear, and gives full details of income and expenditure. One point deserves mention. Under "salaries" the amounts paid to individuals are specified, instead of being set down in one lump sum. Indeed, the whole management of this charity seems to be thorough and enlightened. It may be noted that the out-patients' department, like that of most other hospitals, is yearly increasing.

